# Effect of Early Curing Experiences on Mechanical Properties and Microstructure of ECO-UHPC Prepared by Gold Tailings Sand

**DOI:** 10.3390/ma18040842

**Published:** 2025-02-14

**Authors:** Qi Ouyang, Xianxiang Zhou, Xian Liang, Biao Luo

**Affiliations:** 1 College of Civil Engineering and Architecture, Quzhou University, Quzhou 324000, China; zhouxianxiang1@163.com (X.Z.); liangxian@qzc.edu.cn (X.L.); 2 College of Civil Engineering, Hunan University, Changsha 410082, China

**Keywords:** ultra-high-performance concrete, early curing experiences, fluidity, gold tailings sand, strength, microstructure

## Abstract

Fine gold tailings particles generated from gold mining and refining have the potential to replace high-cost quartz sand in the preparation of economical ultra-high-performance concrete (ECO-UHPC) due to their large stockpiles, low cost, and elimination of grinding. In this study, ECO-UHPC was prepared by substituting quartz sand with gold tailing sand (GTS) at substitution rates of 0%, 25%, 50%, 75%, and 100%. The mechanical properties of ECO-UHPC, including its cubic compressive strength, elastic modulus, and prismatic compressive strength, as well as its leaching toxicity, were experimentally analyzed under various early curing experiences such as ambient-water curing (WC), hot-water curing (HWC), hot-air curing (HAC), and combined curing (CC). Additionally, scanning electron microscopy (SEM) and mercury intrusion porosimetry (MIP) were employed to interpret the macroscopic behavior of ECO-UHPC. The results indicate that the incorporation of waste GTS slightly reduces the fluidity of fresh ECO-UHPC, decreasing it by approximately 6.1% at a full 100% replacement. As a result of waste GTS substitution, the cubic strength of ECO-UHPC experiencing the WC environment during early curing is reduced by 0.7–12.2%. However, the strength of thermally cured ECO-UHPC is comparable to or even higher than that of pure quartz-based G0, with the maximum value occurring in G-50. Specifically, the strength of G-50 cured with HWC, HAC, and CC varies by +20.0%, +40.2%, and +57.7%, respectively, as compared to that of G-50 cured with WC. The evolution of the elastic modulus and the prismatic strength of ECO-UHPC under different early curing conditions and GTS replacement rates aligns closely with that of its cubic strength. In addition, the implementation of thermal curing conditions also limits the leaching of heavy metals from ECO-UHPC, with the best effect observed under CC. This is because appropriate thermal curing promotes the densification of a cementitious substance and the bonding of GTS-cementitious material. Overall, this study demonstrates the feasibility of utilizing waste GTS as a partial or full replacement for quartz sand in ECO-UHPC while maintaining desirable mechanical performance and environmental safety. The findings provide valuable insights into the influence of GTS substitution and early curing regimes on ECO-UHPC properties, highlighting the potential of thermal curing to enhance strength and mitigate leaching risks. Future research should further explore the long-term durability of GTS-based ECO-UHPC and its broader applicability in sustainable construction practices.

## 1. Introduction

UHPC, as a representative advanced building material characterized by high strength and high toughness, has a cubic compressive strength exceeding 120 MPa and a modulus of elasticity between 30 and 60 GPa [1,2]. Due to its high performance and dense particle stacking characteristics, UHPC is widely accepted in many high-quality structures [3,4,5,6]. Currently, the production of UHPC requires a large amount of non-renewable resources such as cement and natural quartz sand (SiO_2_ content ≥ 95%), and the increasing scarcity of natural sand not only affects the stability of the raw material supply but also further increases the material cost of UHPC [7,8,9]. In addition, the collection and grinding of quartz sand, a high-quality aggregate, is delicate and energy-intensive. To improve the high-cost and low-sustainability disadvantages of UHPC, solid wastes such as mechanism sand, coral sand, recycled aggregates, and tailings sand were used as replacement aggregates for the preparation of economical UHPC (i.e., ECO-UHPC) [10,11,12,13]. Recent advancements in the field have shown promising results using various waste materials to enhance the sustainability of concrete composites [14,15]. For instance, Bahmani et al. [16] used steel slag instead of silica sand to prepare environmentally friendly UHPC. It was investigated that the compressive, tensile, and flexural strengths of UHPC with 50% of its content replaced by steel slag were changed by +13.5%, +6.5%, and 20.8%, respectively, in comparison to silica sand-UHPC. Zhang et al. [17] stated that the addition of recycled concrete aggregates damaged the UHPC strength. In recent years, mining and ore smelting have expanded in line with rapid industrialization. As a major gold-producing country, China alone generated 216 million tons of discarded gold tailings in 2018, accounting for 17.84% of the total tailings produced [18]. The disposal of large quantities of low-value tailings, some of which contain heavy metals, not only results in resource wastage but also leads to heavy metal dispersion, polluting the natural environment surrounding storage sites [19,20,21]. Notably, gold tailings, primarily composed of quartz, are ground into fine sand-like particles during the gold extraction process [22]. Therefore, using GTS as a replacement aggregate in UHPC can both mitigate the environmental risks of tailings disposal and reduce the resource and energy consumption associated with obtaining high-quality quartz sand. Certainly, when GTS is incorporated into ECO-UHPC materials, it should be ensured that toxic components do not escape from the matrix. In this context, Ahmed et al. [23] demonstrated that GTS can effectively replace quartz sand in UHPC, and observed that the strength of ECO-UHPC produced with a high content of GTS is not inferior to that of conventional UHPC. Wang et al. [24] explored the use of gold tailings as a partial substitute for cement and aggregates in ECO-UHPC, evaluating its strength and durability. Their findings revealed that the optimal chloride ion permeability of 0.19 × 10^−^^12^ m^2^/s was achieved with a 28% gold tailings substitution.

The exceptional performance of UHPC is not only related to material properties but also the direct influence of the curing environment. Previous research has demonstrated that thermal curing can effectively enhance hydration and the pozzolanic reaction of cementitious components in UHPC, leading to improvements in both the macro- and micro-structural characteristics of the matrix. Currently, the thermal curing conditions that have attracted attention mainly include hot-water curing and dry-heat curing. Tian et al. [25] found that UHPC subjected to 90 °C hot-water curing for 2 days achieved an 18.3% increase in strength compared to ambient curing. Xu et al. [26] reported that dry-heat curing at 180 °C resulted in an impressive compressive strength of 180 MPa for the UHPC at 7 days. In addition to single thermal curing conditions, the performance enhancement of UHPC through combined curing is also noteworthy. Hiremath et al. [27] experimentally demonstrated that a combination of hot-water and dry-heat conditioning led to a 63% increase in the 28-day cubic strength of UHPC compared to ambient-water curing. Despite these advancements, existing studies primarily focus on conventional UHPC, with limited attention given to solid waste-based UHPC, particularly gold tailings-based UHPC (ECO-UHPC). While prior research has confirmed the feasibility of using GTS as a substitute for quartz sand in ECO-UHPC, these investigations have mainly examined the influence of GTS replacement ratios under standard ambient-water curing conditions (20–21 °C). The impact of different early curing conditions, such as hot-water curing, dry-heat curing, and combined curing, on the mechanical properties and microstructure of ECO-UHPC remains largely unexplored. Furthermore, the potential leaching risks associated with GTS incorporation under varying curing conditions have not been systematically evaluated.

To address these research gaps, this study systematically investigates the effects of different early curing experiences on the performance evolution of ECO-UHPC, including cubic strength, prismatic strength, and elastic modulus, by replacing quartz sand with GTS. In addition, heavy metal leaching tests were conducted to assess the environmental safety of ECO-UHPC containing GTS. Furthermore, SEM and MIP analyses were employed to elucidate the microscopic mechanisms governing the macroscopic behavior of ECO-UHPC under different GTS replacement rates and early curing conditions. By clarifying the influence of curing conditions on ECO-UHPC properties, this research provides new insights into optimizing the large-scale utilization of GTS in UHPC production.

## 2. Materials and Test Program

### 2.1. Materials

**Figure 1:**
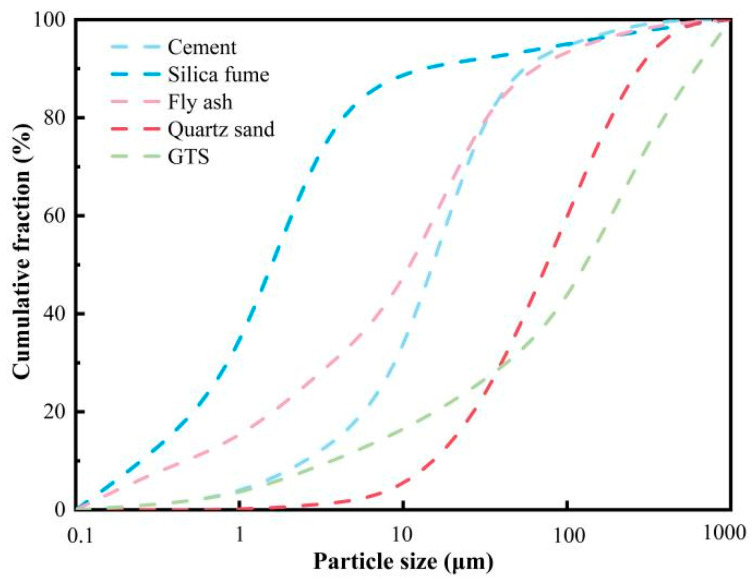
Particle size distribution of powder particles.

The cementitious components primarily include 52.5 grade ordinary Portland cement with a specific surface area of 358 m^2^/kg, silica fume, and Class I fly ash. They are all purchased from Henan Jinrun New Materials Co., Ltd. in Zhengzhou City, China. The detailed oxide contents of these materials are presented in Table 1, based on an X-ray fluorescence (PANalytical Axios, Eindhoven, The Netherlands) analysis. Commercially available quartz sand and screened GTS (maximum particle size not exceeding 1.18 mm) were used as fine aggregates. The particle size distribution of all powder particles is illustrated in Figure 1, derived from a laser particle size analysis (Mastersizer 3000, Malvern City, UK). Copper-coated steel fibers with a diameter of 0.22 mm, a length of 13 mm, and a tensile strength of 2865 MPa were adopted for the physical reinforcement of UHPC. All mixtures were modified with a high-efficiency water reducer, containing 26.0% solid content, to regulate workability.

**Figure 2:**
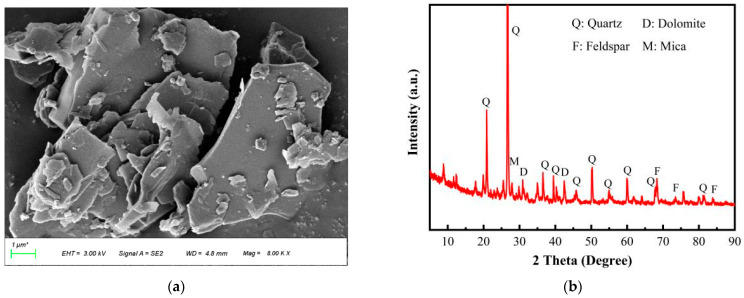
Micro-morphology and mineral characterization of GTS. (**a**) SEM image, (**b**) XRD spectrum.

The GTS was sourced from a gold mine in a mountainous region of China, belonging to the flotation tailing sand, and its micro-morphology is shown in Figure 2a. As observed, the particle shapes of GTS are primarily layered, flaky, or angular. The phase analysis shown in Figure 2b, based on X-ray diffraction (X'Pert PRO MPD, Eindhoven, The Netherlands) evidence, indicates that GTS is primarily composed of silicate minerals, with quartz and feldspar as the main components. Table 1 demonstrates the quantitative characterization results of the oxide contents in GTS. The main component of GTS is SiO_2_ (67.59 wt.%), followed by Al_2_O_3_ (9.10 wt.%), CaO (6.16 wt.%), and other elements, which aligns with the basic characteristics of silicate minerals. It is worth noting that the occurrence environment determines that GTS also contains heavy metal elements. The detailed concentrations of Cd, Pb, As, Zn, and Cu are 0.87 mg/kg, 13.5 mg/kg, 224 mg/kg, 173 mg/kg, and 388 mg/kg, respectively.

### 2.2. Specimen Fabrication

The mix proportions (see Table 2) for the control UHPC and ECO-UHPC, where GTS replaces quartz sand by mass, were designed based on the particle densification theory. The specimen labeled G0 serves as the reference group without the addition of recycled GTS, while G-25, G-50, G-75, and G-100 represent GTS replacement rates of 25%, 50%, 75%, and 100%, respectively.

The cement, silica fume, fly ash, and aggregates (GTS or quartz sand) were thoroughly mixed in advance, followed by the incorporation of water dissolved with a superplasticizer and stirring for 6–8 min, after which steel fibers were evenly dispersed into the mixture until uniform distribution was achieved. After being demolded following 1 day of curing under standard conditions, the specimens underwent various curing treatments. Based on existing studies [28,29], four curing methods were designed: 20 °C water curing (WC), hot-water curing (HWC), hot-air curing (HAC), and combined curing (CC). The detailed curing protocols and procedures are provided in Table 3. After the completion of the above curing treatments, the specimens were moved to the WC environment until testing.

### 2.3. Testing Methods

The workability of freshly mixed ECO-UHPC was evaluated by measuring the spread diameter using the flow table test, following the procedure specified in GB/T 2419-2005 [30]. The test was conducted by placing a standardized amount of the mixture onto a flow table, lifting the mold, and allowing the material to spread freely. The final diameter was recorded as the average of two perpendicular measurements to ensure consistency. The compressive tests on the cubic and prismatic specimens (used to determine the prismatic strength and elastic modulus) of ECO-UHPC were conducted in accordance with the standards outlined in T/CBMF 37-2018 [31], with specimen dimensions of 100 mm × 100 mm × 100 mm and 300 mm × 100 mm × 100 mm, respectively. For the statistical analysis, all experimental results were presented as the mean of at least three independent tests to ensure reproducibility. The reported values represent the mean of all the tested specimens, with standard deviations included to reflect data dispersion. To assess the environmental safety of ECO-UHPC, heavy metal leaching tests were conducted. The leachate was analyzed using inductively coupled plasma mass spectrometry (ICP-MS, model: PerkinElmer 8300, Waltham, MA, USA) to determine the concentration of heavy metals, ensuring compliance with environmental regulations [23]. To explore the micro-mechanisms underlying the effects of GTS replacement and the impact of early curing experiences on ECO-UHPC, small samples were selected from the representative hardened matrix for an SEM (instrument model: Gemini SEM 300, Jena, Germany) and MIP (instrument model: AutoPore V 9600, Norcross, GA, USA) analysis. Additionally, soaking the samples in isopropyl alcohol was implemented as a standard procedure to terminate the hydration of ECO-UHPC. Moreover, immersion in isopropanol before testing was implemented as a routine operation to terminate ECO-UHPC hydration.

## 3. Results and Discussion

### 3.1. Workability

**Figure 3:**
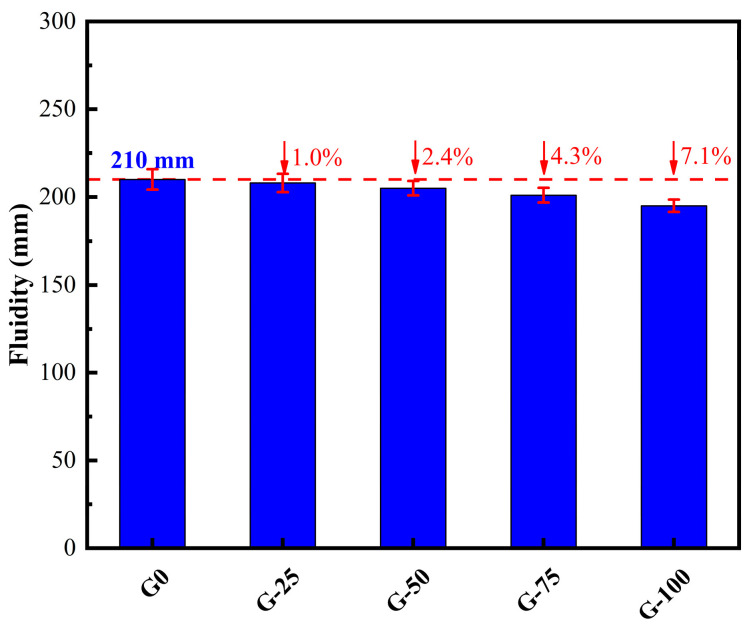
Effect of GTS replacement ratio on the fluidity of UHPC.

The experimental results illustrating the effect of the GTS replacement ratio for quartz sand on the workability of fresh ECO-UHPC are presented in Figure 3. The measured spread diameters for G0, G-25, G-50, G-75, and G-100 are 210 mm, 208 mm, 205 mm, 201 mm, and 195 mm, respectively. As observed, the fluidity of ECO-UHPC exhibits a slight decreasing trend with an increasing GTS replacement ratio, with a maximum reduction of 7.1%. Based on the phy-chemical characteristics of the aggregates, the potential cause of the workability loss with an increasing GTS content may lie in its slightly higher water requirement (88.93%) compared to quartz sand (82.45%). In addition, the flaky morphology of GTS particles exacerbates inter-particle frictional resistance in the context of the low water-to-binder ratio of ECO-UHPC, thereby hindering the free flow of the mixture.

### 3.2. Cubic Compressive Strength

**Figure 4:**
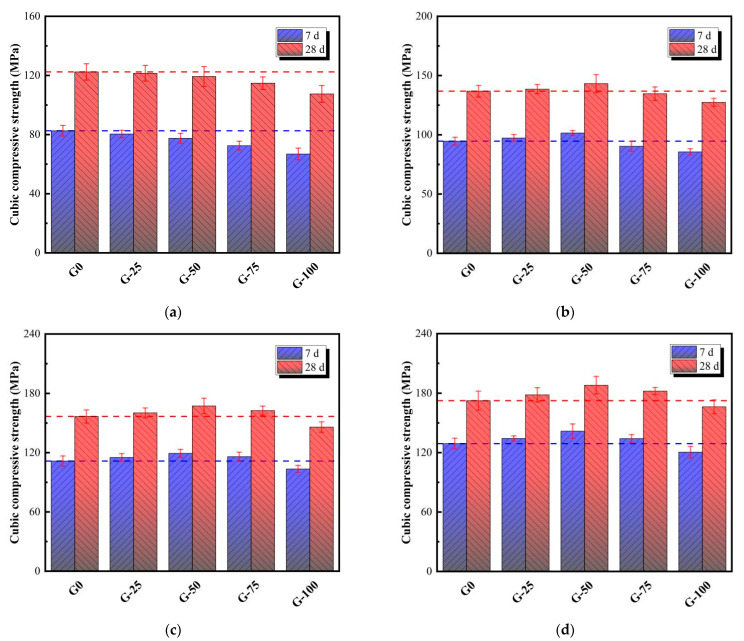
Cubic compressive strength of ECO-UHPC under various early curing experiences: (**a**) WC, (**b**) HWC, (**c**) HAC, and (**d**) CC.

Figure 4 demonstrates the variation in cubic strength of ECO-UHPC containing different GTS replacement rates under four early curing experiences. As shown in Figure 4a, the cubic strength of UHPC decreases gradually with the increasing GTS replacement ratio in the water curing environment at 20 °C. Due to the slightly higher crushing index of GTS (6%) compared to quartz sand (1%), the incorporation of GTS introduces defects into the UHPC matrix, thereby reducing the cubic strength of ECO-UHPC (i.e., G-25/50/75/100) compared to that of quartz sand-based UHPC (G0). This reduction may also be attributed to insufficient bonding between GTS and the cementitious substance [1,32,33]. The 28-day cubic strengths of ECO-UHPC with GTS substitution rates of 25%, 50%, 75%, and 100% are 121.5 MPa, 119.3 MPa, 114.8 MPa, and 107.5 MPa, respectively, which represent reductions of 0.7%, 2.5%, 6.2%, and 12.2% compared to G0. Clearly, when the GTS substitution rate does not exceed 75%, the cubic strength loss of ECO-UHPC is only 0.7–6.2%. However, when GTS completely replaces quartz sand, it has a significant adverse effect on the cubic strength of ECO-UHPC. Notably, the 28-day cubic strength of G-50 undergoing water curing at 20 °C is lower than the 120 MPa threshold defined for UHPC.

The strength of UHPC undergoing different early curing experiences is shown in Figure 4b–d. The thermal curing regime demonstrates beneficial effects on the cubic strength development of ECO-UHPC, which is consistent with the summary of Dong et al. [28]. The 7-day to 28-day strength ratio is used to characterize the early hardening of the ECO-UHPC matrix in this study. The strength ratios of G-0/25/50/75/100 in the WC environment are 67.5%, 66.2%, 65.0%, 63.2%, and 62.2%, respectively, while those in the CC environment are 74.8%, 75.3%, 75.3%, 73.7%, and 72.5%, respectively. Apparently, the thermal curing environment optimizes strength over multiple ages, with the combined curing regime being particularly prominent. This improvement is attributed to the suitable thermal curing, which enhances the hardening efficiency of the composite cementitious system in ECO-UHPC and the interfacial transition zone (ITZ) between GTS and the cementitious materials, thus effectively encapsulating the aggregates to form a dense load-bearing framework [28,34]. After curing in the HWC, HAC, and CC environments, the 28-day cubic strength of G0 increases by 11.8%, 28.0%, and 40.9%, respectively, compared to the WC environment. It is noteworthy that increasing GTS in 20 °C water curing conditions means a decrease in ECO-UHPC strength; however, another phenomenon is presented in the thermal curing experiences, i.e., the strength of ECO-UHPC follows an increase–decrease trend as the GTS substitution rate increases. Regardless of which thermal curing environment (i.e., HWC, HAC, or CC) is used, the highest strength values are obtained for specimen G-50. Specifically, the cubic strength of G-50 in the HWC, HAC, and CC environments is enhanced by 20.0%, 40.2%, and 57.7%, respectively, compared to the WC environment. Additionally, thermal curing appears to offer a better solid waste GTS disposal capacity. Compared to G0, the 28-day strength of G-75/100 in the WC environment changes by −6.2% and −12.2%, respectively, while in the CC environment, the changes are +5.6% and −3.6%.

### 3.3. Elastic Modulus

**Figure 5:**
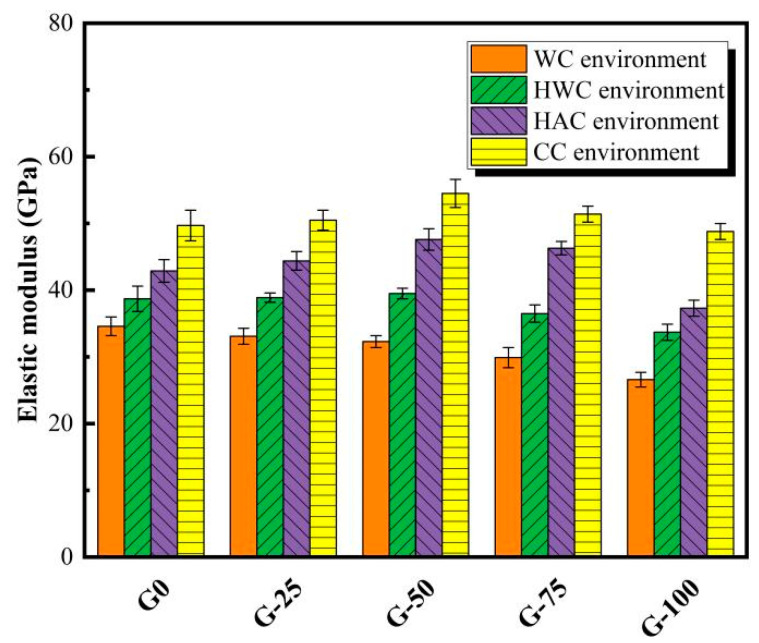
Elastic modulus of ECO-UHPC under various early curing experiences.

Figure 5 demonstrates the variation in the elastic modulus of ECO-UHPC containing different GTS substitution rates under four curing experiences. In this study, the elastic modulus of the fully developed (28-day) matrix is used as a representative result. As presented in Figure 5, the static elastic modulus of the WC-cured ECO-UHPC decreases significantly with the increasing GTS content, which is consistent with the previously observed trend in cubic strength changes. The morphology of GTS particles is characterized by multi-layered composite plates with a lower stiffness (due to the low quartz content and softer minerals), which deform more than quartz sand under compressive stress, thus reducing the modulus values of ECO-UHPC [23]. The elastic modulus of 20 °C water-cured UHPC with GTS substitution rates of 25%, 50%, 75%, and 100% (i.e., G-25/50/75/100) are 33.1 GPa, 32.3 GPa, 29.9 GPa, and 26.6 GPa, respectively, which represent reductions of 4.3%, 6.6%, 13.6%, and 23.1% compared to G0. These changes are slightly larger than the previously observed variations in cubic strength. As for the thermally cured ECO-UHPC specimens, the elastic modulus increases and then decreases with the increasing GTS content, exhibiting a trend that is in good agreement with the evolution of cubic strength. Notably, the specimen G-50 achieves the highest elastic modulus in the HWC, HAC, and CC environments, with values of 39.5 GPa, 47.6 GPa, and 54.5 GPa, respectively. Among these, the combined curing regime induces the optimal modulus values. In contrast, the elastic modulus of G-50 after curing in the CC environment is 68.7% higher than that in the ambient temperature environment. This suggests that the densification of the hardened gel compensates for the matrix defects, and the angular GTS particles, to some extent, limit the relative displacement between the aggregates (GTS or quartz sand) and the cementitious substances, thereby enhancing the deformation resistance of ECO-UHPC [23].

### 3.4. Prismatic Compressive Strength

**Figure 6:**
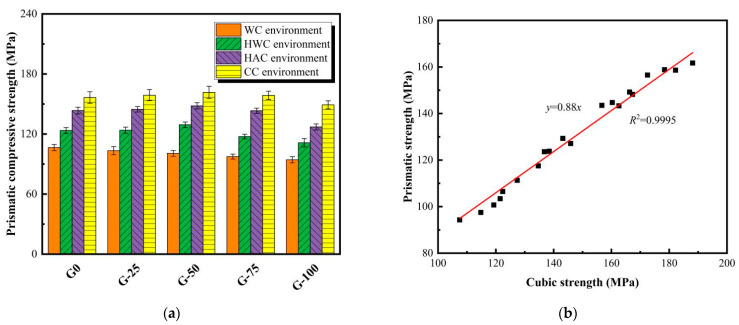
Prismatic compressive strength (**a**) and dimensional effects (**b**) of ECO-UHPC under various early curing experiences.

Figure 6 illustrates the variation in the prismatic strength of ECO-UHPC containing different GTS replacement rates under four curing experiences. In this study, the prismatic strength of the fully developed (28-day) matrix is used as a representative result. As depicted in Figure 6a, the prismatic strengths of G-25/50/75/100 cured in water at 20 °C are 103.4 MPa, 100.7 MPa, 97.5 MPa, and 94.3 MPa, respectively, representing reductions of 2.9%, 5.4%, 8.5%, and 11.5% compared to G0. This indicates that replacing quartz sand with GTS under ambient curing conditions leads to a decrease in the prismatic strength of ECO-UHPC. Similar to cubic strength, different results are observed in ECO-UHPC after a thermal curing treatment. Taking the CC environment, which exhibits the best enhancement effect, as an example, the prismatic strengths of G-25, G-50, G-75, and G-100 are 158.8 MPa, 161.7 MPa, 158.6 MPa, and 149.2 MPa, respectively, with changes of +1.5%, +3.3%, +1.3%, and −4.7% compared to G0. It is clear that ECO-UHPC incorporated with GTS can reach or even exceed the mechanical strength of G0 with an appropriate initial curing treatment. This is because thermal curing compensates for the weak bonding between GTS and the cementitious substances, thereby improving the tolerance of the UHPC matrix to substitute aggregates. As shown in Figure 6b, the ratio of prismatic to cubic strength for ECO-UHPC at 28 days is 0.88, indicating a certain size effect.

### 3.5. Inhibition of Heavy Metal Leaching by UHPC

Table 4 presents the leaching concentrations of the heavy metal ions in ECO-UHPC at 28 days. The concentrations of the zinc and copper ions gradually increase with the increasing waste GTS content. When the GTS replacement rate reaches 50%, the leaching concentrations of the zinc and copper ions are 0.215 mg/L and 0.065 mg/L, respectively. When quartz sand is fully replaced by GTS in ECO-UHPC, their concentrations rise to 0.262 mg/L and 0.081 mg/L, respectively. Nevertheless, all the measured concentrations remained significantly below the permissible thresholds established by various environmental protection regulations, confirming the environmental safety of ECO-UHPC that incorporates high GTS dosages. The primary sources of hazardous elements in the leachate are residual sulfide minerals, such as pyrite and arsenopyrite, present in gold tailings sand. These minerals can undergo oxidation and release heavy metal ions under acidic conditions [35]. However, the relatively low leaching concentrations observed in this study can be attributed to the limited solubility of heavy metals in their oxidized state and the highly dense matrix of ECO-UHPC, which restricts ion migration [23,36,37]. Furthermore, a comparison of the ion concentrations of G-50 under the four curing environments reveals that the leaching of heavy metals from ECO-UHPC is further reduced after thermal curing, especially under the combined curing regime. This suggests that early thermal curing plays a crucial role in enhancing the hydration degree and refining the pore structure of ECO-UHPC. In other words, the dense ECO-UHPC matrix effectively stabilizes the heavy metal ions and blocks the ion migration channels, thereby providing a new scientific approach for the re-utilization of waste GTS [37].

### 3.6. SEM Examination

**Figure 7:**
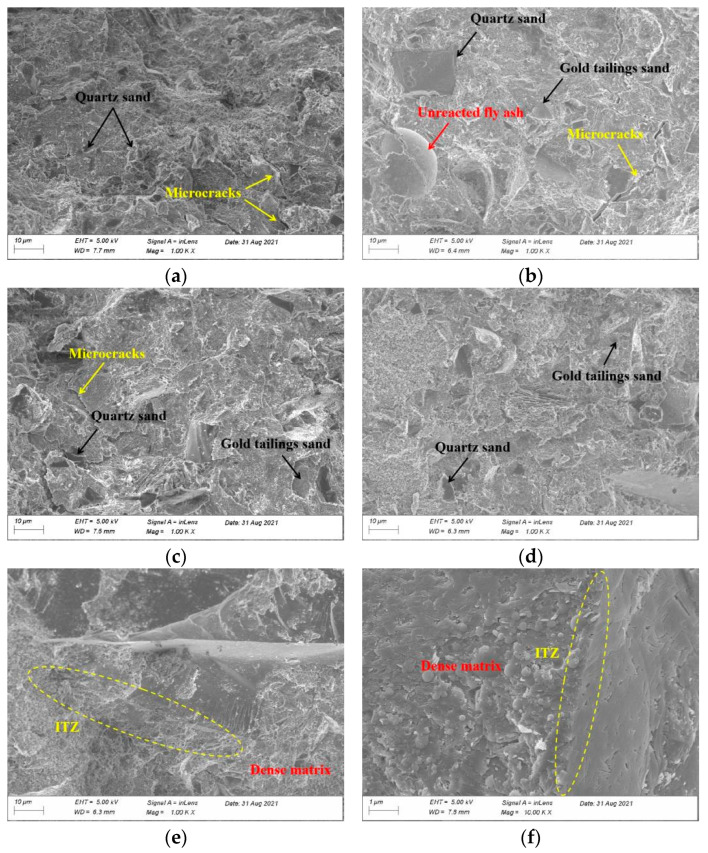
Effect of GTS replacement ratio and early curing experiences on the microstructure of 28 d cured ECO-UHPC: (**a**) G0 (WC); (**b**) G-50 (WC); (**c**) G-50 (HWC); (**d**) G-50 (HAC); (**e**) G-50 (CC); and (**f**) G-50 (CC) at 1 μm.

To further investigate the impact of curing experiences and GTS substitution rates on the micro-mechanisms of ECO-UHPC, the WC-cured G0 and G-50, as well as the HWC-, HAC-, and CC-cured G-50 were selected as representative samples for the analysis. As observed in Figure 7a,b, both the G0 and G-50 specimens under 20 °C water curing exhibit a few defects characterized by microcracks, primarily located near the joints between the hardened paste and the aggregates (quartz sand and GTS). This may be the microscopic reason for limiting the high strength of ambient-cured ECO-UHPC [28]. In addition, defect growth due to GTS substitution is not clearly detected in all the microscopic evidence. Dong et al. [28] found that high-temperature or autoclaving treatments are beneficial for transforming the low hydration of cement in UHPC, and may also stimulate the reactivity of materials such as silica fume, fine quartz, or fine tailings. A comparative observation of the microstructures of the G-50 specimens cured under WC, HWC, HAC, and CC conditions (Figure 7b–e) shows that all the thermal curing experiences enhance the densification of the ECO-UHPC matrix to varying degrees, as compared to ambient temperature curing. This confirms at the microscopic level that thermal curing experiences provide a foundation for the strength enhancement of ECO-UHPC. The microcracks extending from the transition zone of the aggregate-hardened paste and the large unreacted fly ash particles observed in the WC-cured G-50 specimen are not found in the thermal-cured specimens. The implementation of thermal curing optimizes the hydration and pozzolanic reactions of the ECO-UHPC, fundamentally altering the densification of the hardened matrix [29]. As the curing environment shifts to CC, there are no visible micro-defects in the G-50 (CC) sample shown in Figure 7e, and the aggregates are fully embedded and encapsulated by the cementitious substance. Furthermore, under higher magnification (Figure 7f), it is observed that the joints between the hardened paste and the aggregates in the G-50 (CC) sample exhibit a blurred interface with strong bonding. In addition, the GTS with the optimal substitution content demonstrates a good filling effect on the ECO-UHPC matrix. This microstructural enhancement is the underlying reason for the improved strength of the thermal-cured ECO-UHPC. Interestingly, the CC environment appears to be more effective at enhancing the mechanical strength of the hardened matrix than the single thermal curing environments (HWC and HAC). This is because the first stage of combined curing, which involves hot-water curing, promotes hydration and forms a relatively dense load-bearing framework in ECO-UHPC. Subsequently, dry-heat curing, aided by the steam pressure of internal water, creates an autoclave-like environment, which stimulates the continued hydration of residual cementitious particles and the pozzolanic reaction of the additives [28,29].

### 3.7. Pore Structure Analysis

**Figure 8:**
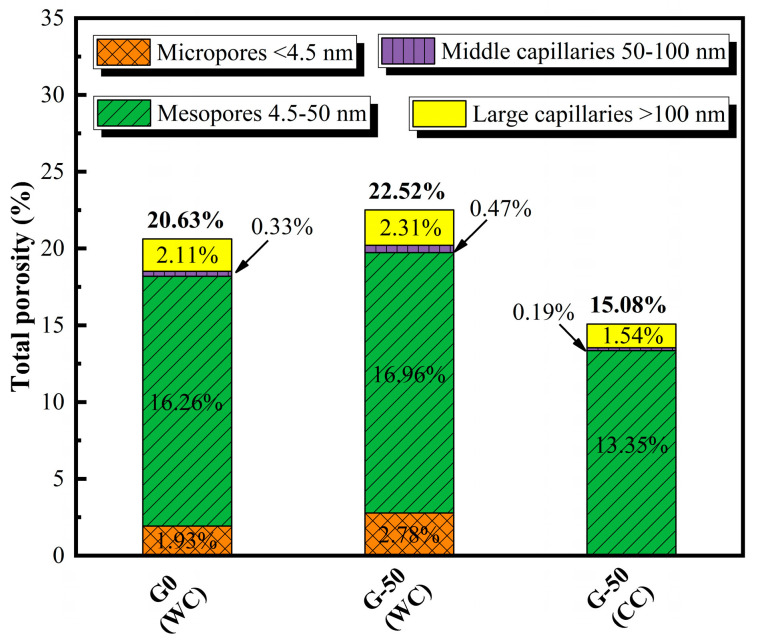
Total porosity and pore size distributions of ECO-UHPC containing GTS.

The pore characteristics of G0 and G-50 when experiencing the WC environment, as well as G-50 when experiencing the CC environment, are shown in Figure 8. To better distinguish the porosity characteristics of ECO-UHPC under different GTS replacement rates and early curing experiences, the pore sizes are classified into four ranges: micropores (<4.5 nm), mesopores (4.5–50 nm), middle capillaries (50–100 nm), and large capillaries (>100 nm) [40]. As demonstrated in Figure 8, the total porosity of G0 (WC), G-50 (WC), and G-50 (CC) are 20.63%, 22.52%, and 15.08%, respectively. Increasing the GTS substitution rate results in a slightly higher total porosity of the ECO-UHPC than the pure quartz sand-based UHPC in the 20 °C water curing environment. Moreover, the main pore deterioration detected is due to the increase in micropores with a size smaller than 4.5 nm. This implies that the bonding performance of waste GTS with the hardened paste is weaker than that of quartz sand. A comparative observation of the pore structure of G-50 (WC) and G-50 (CC) reveals that thermal curing significantly reduces the total porosity of ECO-UHPC, with a comprehensive refinement of all types of pores. Specifically, the porosity of the micropores, mesopores, middle capillaries, and large capillaries decreases from 2.78%, 16.96%, 0.47%, and 2.31% in the G-50 (WC) sample to 0%, 13.35%, 0.19%, and 1.54% in the G-50 (CC) sample, respectively. It should be noted that the “autoclave reaction” created by the combined curing method optimizes the densification process of the binder phase, thereby repairing the weak bonding between the waste GTS and hardened paste. This improves the mechanical strength of ECO-UHPC and enhances its ability to dispose of waste GTS [28,29].

## 4. Conclusions

In this research, the fluidity of ECO-UHPC prepared by replacing quartz sand with waste GTS was first investigated, followed by the effects of the GTS replacement ratios (0, 25%, 50%, 75% and 100%) and early curing experiences (WC, HWC, HAC and CC) on the mechanical strength of ECO-UHPC. Finally, the micro-mechanisms were analyzed using SEM and MIP. The primary conclusions are as follows:

(1) The fluidity of fresh ECO-UHPC decreases slowly with the increasing GTS substitution rate as influenced by water demand and the particle morphology of the waste GTS.
(2) The cubic strength of the WC-cured ECO-UHPC gradually decreases with the increase in the GTS substitution rate, with a reduction range of 0.7–12.2%. However, after thermal curing, the strength of the ECO-UHPC shows an increase–decrease trend with the increase in GTS content, achieving the optimal strength at G-50. The cubic strength of G-50 in the HWC, HAC, and CC environments increases by 20.0%, 40.2%, and 57.7%, respectively, compared to the WC environment. It is noteworthy that G-75/100 with high substitution rates obtain a strength comparable to or even higher than G0 under thermal curing conditions, indicating that thermal curing optimizes the performance of ECO-UHPC and enhances its ability to accommodate waste GTS.
(3) The elastic modulus of the WC-cured G-25/50/75/100 decreases by 4.3%, 6.6%, 13.6%, and 23.1%, respectively, compared to G0. In contrast, in the optimal thermal curing environment (i.e., CC), the elastic modulus of G-25/50/75/100 are closer to that of G0, with a change ranging from −1.8% to 9.7%. Additionally, the prismatic strength of ECO-UHPC is found to be linearly correlated with the cubic strength.
(4) The main heavy metals leached from ECO-UHPC are zinc and copper, and their concentrations gradually increase with the GTS substitution rate. However, they remain well below the allowable threshold set by the established standards. The concentration of heavy metals is significantly reduced after the early thermal treatment of ECO-UHPC.
(5) Microscopic observation and the pore structure analysis show that increasing the GTS substitution rate slightly increases the porosity of ECO-UHPC. Thermal curing promotes the densification of the cementitious materials, which compensates for the weak bonding between GTS and the cementitious materials, thereby improving the strength of ECO-UHPC and blocking the migration pathways of heavy metals. This improvement effect is most evident with combined curing, due to the “autoclave effect” that stimulates further hydration and pozzolanic reactions of the residual cementitious particles.

Future research should focus on the long-term durability of ECO-UHPC under harsh environments, optimizing the mix design to improve workability and strength, assessing its economic viability for large-scale applications, and conducting comprehensive environmental evaluations to ensure the sustainable utilization of waste GTS.

## Figures and Tables

**Table 1:** Oxide contents (wt.%) of raw materials.

Type	CaO	K_2_O	MgO	Na_2_O	Al_2_O_3_	Fe_2_O_3_	SiO_2_	Others	LOI
Cement	68.35	0.82	0.91	0.17	2.68	2.35	23.14	1.58	2.12
Fly ash	5.47	1.96	0.79	2.10	32.53	8.79	44.26	4.10	3.46
Silica fume	0.32	0.85	0.51	0.12	0.27	0.15	93.55	4.23	2.26
GTS	6.16	1.95	1.46	0.93	9.10	3.35	67.59	9.46	5.37
Quartz	0.01	0.02	0	0.01	0.05	0.01	99.86	0.04	-

**Table 2:** Mixture proportions of the designed UHPC (kg/m^3^).

Samples	Cement	Silica Fume	Fly Ash	Quartz Sand	GTS	Steel Fiber	Water	Water Reducer
G0	786	220	140	966	-	158	207	23
G-25	786	220	140	724	242	158	207	23
G-50	786	220	140	483	483	158	207	23
G-75	786	220	140	242	724	158	207	23
G-100	786	220	140	-	966	158	207	23

**Table 3:** Detailed early curing experiences and process.

Curing Condition	Identification	Curing Process
20 °C water curing	WC	Maintained in water at 20 °C for 28 days
Hot-water curing	HWC	Maintained in water at 90 °C for 2 days
Hot-air curing	HAC	Maintained in dry-heat air at 250 °C for 3 days
Combined curing	CC	Maintained in hot water at 90 °C for 2 days, followed by heating in dry air at 250 °C for 3 days

**Table 4:** Concentration of heavy metal leaching in ECO-UHPC (mg/L).

Heavy Metal	G0 (WC)	G-50 (WC)	G-100 (WC)	G-50 (HWC)	G-50 (HAC)	G-50 (CC)	GB 16889-2008 [38]	GB 5085.7-2019 [39]
Zn	0.152	0.215	0.262	0.201	0.197	0.158	100	100
Cu	0.020	0.065	0.081	0.066	0.063	0.044	40	100
As	<0.005	<0.005	<0.005	<0.005	<0.005	<0.005	0.3	5
Pb	<0.005	0.011	0.024	<0.005	<0.005	<0.005	0.25	5
Cd	<0.001	<0.001	<0.001	<0.001	<0.001	<0.001	0.15	1

## Data Availability

The raw data supporting the conclusions of this article will be made available by the authors on request.
